# Recent advances in radionuclide medical imaging techniques

**DOI:** 10.3389/fmed.2025.1662020

**Published:** 2025-12-03

**Authors:** Shuyu Xu, Ge Liu, Qingyang Wei, Hui Liu, Jing Wu, Yaqiang Liu, Zuo-Xiang He

**Affiliations:** 1 Beijing Engineering Research Center of Industrial Spectrum Imaging, School of Automation and Electrical Engineering, University of Science and Technology Beijing, Beijing, China; 2 Department of Engineering Physics, Tsinghua University, Beijing, China; 3 Center for Advanced Quantum Studies, School of Physics and Astronomy, Beijing Normal University, Beijing, China; 4 Department of Nuclear Medicine, Beijing Tsinghua Changgung Hospital, Beijing, China

**Keywords:** PET, SPECT, radionuclide, imaging technique, cascade gamma photon imaging

## Abstract

Radionuclide imaging combines nuclear technique and medicine through the administration of radioactive drugs into living organisms, followed by imaging with specialized instruments. This technique is essential in modern medicine, facilitating diagnosis, treatment, medical research, exploration of drug mechanisms, and evaluation of drug efficacy. This review critically synthesizes the pivotal advancements in the field over the past decade, moving beyond a descriptive overview to analyze the clinical impact and translational barriers of emerging technologies. We evaluate key innovations in traditional modalities, such as the role of CZT detectors in transforming cardiac SPECT and the impact of TOF and DOI on quantitative accuracy in PET. Furthermore, we provide a comparative analysis of multimodal systems (e.g., PET/CT vs. PET/MRI), focusing on their clinical decision-making context. Emerging paradigms like self-collimation and cascade gamma photon imaging are examined as potential solutions to the inherent limitations of current systems, with a critical assessment of their technology readiness levels. A significant focus is placed on the rapidly evolving landscape of theranostics, highlighting the synergy between imaging and targeted radionuclide therapy. By identifying key trends, persistent challenges, and future directions, this review provides a comprehensive and critical perspective on the ongoing evolution of radionuclide imaging from a technological and clinical standpoint.

## 1 Introduction

Radionuclide imaging, a critical intersection of nuclear technique and medicine, is an essential tool for visualizing specific molecular-level changes in living tissues, cells, and subcellular structures. Characterized by high sensitivity, specificity, and quantifiability, this technique enables observation of metabolic activities, blood perfusion, neural functions, cell receptors, and protein expression in specific organs or tissues. It aids in the diagnosis, staging, and monitoring of tumor progression, as well as in assessing neurological, cardiovascular, coronary artery disease (1), and brain disorders (2), providing essential evidence for early diagnosis and targeted treatment. It also monitors drug absorption, distribution, metabolism, and excretion to evaluate therapeutic effects, perform dosimetry analysis, and facilitate drug development.

Radionuclide imaging operates on the principle of administering radiopharmaceuticals into the living organism, which are circulating within the body and accumulate differently in normal and diseased tissues. As physiological activities such as organ metabolism occur, a three-dimensional dose distribution is formed within the body; the emitted gamma rays are detected, and an image of the 3D distribution of the radiopharmaceuticals is reconstructed (3).

The most commonly used radionuclide imaging medical techniques include gamma camera, single photon emission computed tomography (SPECT), positron emission tomography (PET) and multimodality imaging techniques such as SPECT/CT, PET/CT and PET/MRI.

However, despite their widespread clinical success, conventional modalities like SPECT and PET face persistent limitations. SPECT’s reliance on mechanical collimators severely compromises sensitivity for spatial resolution. PET, while more sensitive, still contends with challenges like the parallax effect and detector timing limits, which impact resolution and image quality. This fundamental trade-off between sensitivity and spatial resolution in both systems motivates the need for technological advancements.

This paper focuses on the recent advancements in radionuclide imaging, a rapidly growing field that has attracted substantial attention from research institutions globally. It aims to summarize the latest research achievements in this area over the past decade, covering a range of imaging techniques.

## 2 Research progress of traditional radionuclide imaging techniques

The advent of the scanner in the 1950s marked the inception of radionuclide imaging (4). A pivotal advancement occurred in 1964 with the introduction of the first scanner to utilize a single crystal scintillation detector (5). This innovative device, endowed with the ability to delineate spatial distribution and temporal resolution, facilitated rapid imaging and the capture of dynamic physiological processes, known as the gamma camera or scintillation camera. Since the 1960s, gamma camera technology has progressed substantially, with recent developments focusing on portable intraoperative gamma cameras (IGCs). These devices provide real-time, high-resolution 2D imaging during surgery, offering significant advantages over conventional 1D gamma probes. Advances in detector materials, collimator geometries, and readout architectures have collectively enhanced the spatial resolution, sensitivity, and clinical versatility of IGCs, with hybrid imaging capabilities further supporting intraoperative decision-making (6).

Technological advancements have markedly enhanced the performance of radionuclide imaging devices, leading to superior image quality and more precise disease identification, localization, and tracking. Key performance indicators for these devices include spatial resolution, sensitivity, noise equivalent count rate, timing resolution, and energy resolution. An ideal imaging system should feature low dead time, low scatter fraction, high counting capability, high energy resolution, and a high signal-to-noise ratio. Most importantly, it must maintain high sensitivity and spatial resolution, although these two factors often trade off against each other (7). The overarching goal of radionuclide imaging development is to augment both device sensitivity and spatial resolution to attain higher-quality images and more accurate diagnostic information.

### 2.1 SPECT

Single-Photon Emission Computed Tomography (SPECT) is a type of ECT technology specifically designed for tomographic imaging of radiopharmaceuticals such as ^99m^Tc (Technetium), ^131^I (Iodine), ^111^In (Indium), and ^201^Tl (Thallium), which emit single gamma photons during each decay event (8, 9). Its cost-effectiveness and independence from expensive cyclotron support have enabled SPECT to achieve widespread clinical application (10). However, the development of SPECT technology has long been constrained by two fundamental physical limitations: sensitivity loss caused by mechanical collimation and the intrinsic spatial resolution limits imposed by detector materials (11). These constraints result in significant partial volume effects, reducing image quantitative accuracy and potentially leading to the omission of small, early-stage lesions. To overcome these limitations, recent research on high-performance SPECT has primarily focused on two major directions: revolutionary advancements in detector technology and innovative system geometry design.

The detector serves as the core component of a SPECT system, and innovations in detector materials represent the fundamental driving force behind improved imaging performance. Traditionally, clinical SPECT detectors mainly utilize large-volume continuous thallium-doped sodium iodide (NaI(Tl)) crystals (typical size 500 × 400 × 9.5 mm^3^) coupled with arrays of PMTs (12). However, traditional large-volume NaI(Tl) crystals have poor intrinsic spatial resolution (3 ~ 5 mm@140 keV). Although efforts to improve spatial resolution have been made, such as the work by Rozler et al. (13), who employed compact pixelated detectors with a pixel size of 2.75 × 2.75 × 10 mm^3^ in clinical SPECT systems, the hygroscopic nature of NaI(Tl) crystals complicates the fabrication process, thereby limiting further advancements in detector miniaturization.

To overcome the performance bottlenecks of traditional detectors, various novel scintillator materials have emerged. The advent of cadmium zinc telluride (CZT) semiconductor detectors marked a paradigm shift in SPECT technology (14–18), offering higher energy resolution, enhanced sensitivity, and a more compact structure (19, 20). By directly digitizing photons, these detectors achieve improved sensitivity and spatial resolution, with pixel size in D-SPECT reaching 2.46 × 2.46 × 5 mm^3^ (14). This technological breakthrough has directly led to the development of a new generation of high-performance SPECT systems, such as GE’s Discovery NM/CT 870 CZT (21) and Switzerland’s Veriton-CT SPECT/CT (22), both of which deliver faster acquisition speeds and superior energy discrimination (23). Clinical evidence further demonstrates that CZT-SPECT supports low-dose, high-temporal-resolution gated nuclear cardiology imaging (24, 25), enhancing the accuracy and reproducibility of quantitative cardiac functional parameters such as ejection fraction and filling rate (26). Moreover, its excellent energy resolution provides a foundation for advanced applications like multi-isotope imaging. However, the high manufacturing cost of CZT crystals remains a major barrier to widespread adoption, making cost-effectiveness a critical factor in clinical decision-making.

Between NaI(Tl) and CZT, researchers have been actively exploring alternative materials that offer a more optimal balance between performance and cost. For instance, lanthanum bromide (LaBr₃) provides excellent light output and energy resolution (6%@140 keV), yet its intrinsic background radiation and hygroscopic nature present practical challenges for clinical application. In recent years, cerium-doped gadolinium aluminum gallium garnet (GAGG) has become a popular scintillation crystal for SPECT due to its high light yield, fast decay time, high attenuation capability, lack of background radiation, and non-hygroscopic properties (27–30).

Regardless of the material used, the design of SPECT detectors has always faced a fundamental physical trade-off between sensitivity and spatial resolution. To ensure sufficient detection efficiency (sensitivity), the scintillation crystal must possess a certain thickness; however, increasing the thickness inevitably enhances light scattering or charge diffusion, thereby degrading spatial resolution. Although technologies such as micro-columnar CsI crystals can achieve exceptionally high resolution, their limited thickness results in poor detection efficiency (31–33). Consequently, optimizing the selection among different crystal materials—such as GAGG, LaCl_3_, CsI, LaBr_3_, and YAlO_3_—based on their physical characteristics for specific clinical applications represents a key challenge in the design of next-generation SPECT systems (34).

In parallel with innovations in detector materials, optimizing system geometry to capture photon information more efficiently represents another critical avenue for enhancing SPECT performance. Multi-pinhole (MPH) collimation was originally introduced as an ingenious engineering strategy to compensate for the limited resolution of conventional detectors by leveraging the magnification effect of pinholes. This approach has achieved remarkable success in small-animal imaging, reaching submillimeter spatial resolution. For instance, an advanced multi-pinhole collimator small animal SPECT system (U-SPECT5/CT) developed at Delft University of Technology (35, 36) achieved a spatial resolution of 0.25 mm and a sensitivity of 0.038%, albeit with an effective field of view(FOV) of only about 1 cm^3^. In recent years, this concept has been extended to clinical brain SPECT (37) and whole-body SPECT (38) imaging, where it has been combined with ring detector configurations to achieve spatial resolutions of 2–3 mm—an improvement of 4–5 times over traditional SPECT systems. However, this magnification-based strategy also introduces new constraints: the use of large, low-resolution detector modules limits system compactness and, consequently, the number of pinholes that can be integrated, thereby restricting further improvements in overall system sensitivity. However, this magnification-based strategy also introduces new constraints: the use of large-volume low-resolution detector modules limits system compactness and the number of pinhole collimators that can be integrated, thereby restricting further improvements in overall system sensitivity.

In contrast to the aforementioned strategy, another design approach combines ultra–high-resolution detectors with a pinhole reduction imaging scheme (31, 39, 40). This approach minimizes detector size, enabling a more compact system design that can accommodate a greater number of detectors within a limited space, thereby directly enhancing overall system sensitivity. For example, the MRC-SPECT-II system leverages the exceptionally high intrinsic resolution of CZT detectors (approximately 100 μm) to support a complex collimator design incorporating 1,536 ultrafine pinholes (39). Similarly, the FastSPECT-III system achieves high system sensitivity within a 15 mm diameter imaging space by integrating 20 gamma cameras (41). At the frontier of SPECT innovation, Laue lens diffraction imaging technology explores an entirely new physical principle, aiming to eliminate the need for conventional collimators and achieve a spatial resolution down to 0.113 nL (42).

The leap in SPECT performance is not an isolated technological advancement but rather the result of the synergistic evolution among hardware innovation, advanced algorithms, and clinical demands. Improvements in hardware resolution must be complemented by sophisticated image reconstruction algorithms capable of accurately correcting physical effects—such as partial volume correction and dosimetry (43)—and leveraging deep learning–based optimization techniques. Only through this integration can the full potential of modern SPECT systems be realized, enabling more precise diagnostic and therapeutic applications in cardiology, oncology, and neurology (44).

In addition, specialized geometric designs can also enhance the spatial resolution and sensitivity of SPECT systems (45, 46). These designs facilitate more efficient collection of gamma rays from the target. For instance, InSPira HD employs a ring-shaped detector system utilizing NaI(Tl) scintillators paired with adaptive collimators, achieving a spatial resolution of 6–8 mm full width at half maximum (FWHM).

This synergistic advancement in both hardware and software ultimately serves to deepen clinical applications. For instance, the superior energy resolution of modern detectors renders complex applications, such as dual-tracer imaging, more reliable. Examples include stress and rest myocardial perfusion imaging with ^99m^Tc (140 keV) and ^201^Tl (75 keV/167 keV), as well as neurotransmitter agents labeled with ^123^I (159 keV). By acquiring data from different energy windows simultaneously, this approach not only shortens examination times but also ensures the perfect spatiotemporal registration of multiple physiological data sets, although crosstalk correction between the energy windows remains a primary focus of current research. In summary, breakthroughs in material science, ingenuity in system design, and enhanced computational power are collectively propelling SPECT towards higher precision, greater speed, and improved quantitative capabilities. This evolution is opening new frontiers for its theranostic applications in fields such as cardiology, oncology, and neurology.

### 2.2 PET

Positron Emission Tomography (PET) is a form of ECT that utilizes positron-emitting radioactive isotopes, including ^11^C, ^13^N, ^15^O, ^18^F, ^64^Cu, and ^82^Rb (47). PET employs coincidence detection technology, which eliminates the need for mechanical collimators. During imaging, positron-emitting nuclei decay within the biological source and release positrons. These positrons undergo annihilation, producing a pair of gamma photons, each with an energy of 511 keV and traveling in opposite directions. Within a designated time window (typically 0–15 ns), the system detects pairs of gamma photons at 180° (±0.25°) to each other, identifying them as coincidence events. The detected positions of the two gamma photons in a coincidence event are connected to generate a line of response (LOR), indicating the location of the radioactive isotope. This method is also known as electronic collimation. However, during the imaging process, PET is subject to various factors such as Compton scattering in the object, leading to discrepancies between the collected data and the actual conditions, resulting in image quality distortion (48). Therefore, the development of PET technology is, in essence, a relentless pursuit of higher spatial and temporal resolution, aiming to approximate as closely as possible the true spatiotemporal coordinates of the annihilation events.

The foundation of precise detection lies in the choice of detector crystal material, which directly determines the ultimate performance limit of a PET system. The choice of detector crystal material significantly affects the performance of PET equipment. PET detector crystals require high density and atomic number to achieve high stopping power, rapid rise and decay times for improved timing resolution, and high light output for enhanced energy resolution, along with properties such as high mechanical strength and moisture resistance. From the early use of NaI to the mainstream adoption of bismuth germanate Bi_4_Ge_3_O_12_ (BGO) and cerium-doped gadolinium oxyorthosilicate Gd_2_SiO_5_(Ce) (GSO), and further to the currently prevalent cerium-doped lutetium oxyorthosilicate Lu_2_SiO_5_(Ce) (LSO) and cerium-doped lutetium-yttrium oxyorthosilicate LYSO: Ce (LYSO) crystals, each iteration of detector materials has sought an optimal balance among these critical performance parameters. In recent years, fully digital PET detectors based on cadmium zinc telluride (CZT) have become a research hotspot (49). Unlike conventional designs, they eliminate the need for scintillators and photoconversion, offering the potential for fundamental improvements in both energy and spatial resolution. However, every choice entails compromise—no perfect crystal yet exists. Thus, the selection of detector materials remains a complex trade-off among equipment performance, manufacturing cost, and the specific requirements of clinical applications.

Beyond material selection, the physical segmentation of crystals represents the most direct approach to improving spatial resolution. By dividing the crystal into smaller elements, higher signal-to-noise ratio(SNR) can be achieved. For instance, the detector crystal unit size of the microPET scanner is approximately 1.5 mm, yielding a resolution of 1.5–1.75 mm. Taking this concept further, finely segmented scintillation crystals can be stacked longitudinally and coupled with high-pixel photodetectors to track the interaction points of photons within the crystal. This enables three-dimensional spatial localization and establishes the foundation for higher-dimensional correction techniques.

If the optimization of crystal materials represents a “quantitative” enhancement in PET performance, then the introduction of Time-of-Flight (TOF) (50) technology marks a true “qualitative” leap. The principle of TOF is based on the fact that the path length from the annihilation point to the detector varies for each coincident photon, resulting in different arrival times for individual gamma rays. TOF-PET utilizes the time difference of arrival of annihilation photons at the detector to confine the positron emission location to a small segment along the LOR connecting two scintillation crystals. In conventional PET image reconstruction, algorithms must distribute the probability of each detected event uniformly along the entire LOR, which inevitably amplifies the uncertainty of individual events and propagates it across the entire image—becoming a major source of noise. TOF technology, by dramatically reducing this region of uncertainty, significantly enhances the SNR. This improvement in SNR yields substantial clinical benefits: images of equivalent quality can be obtained in a shorter acquisition time, higher-quality images can be achieved within the same scan duration, and in some cases, the administered radiopharmaceutical dose can even be reduced.

In the early stages, the limited timing resolution of conventional photomultiplier tubes (PMTs) and scintillation crystals—typically greater than 1 ns—confined TOF technology to a theoretical concept for many years. It was not until the advent of fast-decay crystals such as LSO and LYSO, followed by breakthroughs in photodetector technology, that timing resolutions on the order of several hundred picoseconds became achievable (51). As a result, TOF technology successfully transitioned from laboratory research to clinical application. The initial batch of commercial TOF-PET scanners, such as Philips’ 3D PET scanner (Ingenuity TF) (52), featured timing resolutions between 500 and 600 ps. To further enhance quantitative accuracy, recent reconstruction frameworks have incorporated simultaneous activity and attenuation estimation (SAA) into the TOF-PET model. Methods based on biconvex optimization with total variation constraints have improved stability and image quality over traditional approaches, enhancing TOF-PET quantification under clinical conditions (53).

Pushing TOF timing to the tens of picosecond level has enabled a revolutionary paradigm: reconstruction-free PET. A recent study by Kwon et al. demonstrated this by achieving a 32 ps timing resolution using prompt Cerenkov radiation, a novel integrated detector (CRI-MCP-PMT), and a CNN. This precision is sufficient to directly localize annihilation events (~4.8 mm spatial precision), entirely bypassing tomographic reconstruction. This new modality, termed dPEI, promises to transform PET system design by eliminating the geometric and sampling constraints of traditional reconstruction (54).

The utilization of Silicon Photomultipliers (SiPMs) has further enhanced the performance of TOF-PET (55). SiPMs exhibit excellent internal timing resolution, thereby improving the sensitivity, image resolution, and maximum count rate of TOF-PET (56, 57). The TOF timing resolution of SiPMs systems surpasses 200 ps. To achieve a timing resolution of 100 ps, a new detector design approach involves mounting SiPMs on the long edge of crystals instead of the traditional end-to-end installation (58). Additionally, compared to the significantly larger PMTs used in traditional PET systems, SiPMs or Avalanche Photodiodes (APDs) (59) enable direct one-to-one correspondence between the photodetector and the detector unit. This facilitates coupling with smaller crystal elements, leading to a spatial resolution improvement in PET. For example, United Imaging has improved the spatial resolution of PET to less than 2.35 mm. Furthermore, SiPMs and APDs are immune to electromagnetic interference, making them suitable for PET/MRI multimodal imaging.

While advancements in SiPMs are pushing timing resolution towards the 100 ps frontier, researchers are simultaneously exploring novel physical mechanisms to push far beyond this barrier. Two emerging technological routes are particularly prominent: Cherenkov radiation-based detection and liquid noble gas scintillation technology. The former approach utilizes the prompt Cherenkov photons generated by charged particles within crystals like BGO. Although this method faces challenges due to a low and stochastic photon yield, experimental detectors have achieved an ultra-fast timing resolution of approximately 30 ps, which could enable novel scanner geometries such as dual-panel PET systems (60, 61). The latter route is exemplified by the use of xenon-doped liquid argon (LAr + Xe), which offers a scintillation time about seven times faster and a 25% higher photon yield compared to LYSO (62). By doping with xenon, this technology overcomes the inherent issues of pure LAr, such as a slow decay component and a mismatched emission wavelength, thereby significantly improving light detection efficiency and the system’s ability to handle high event rates. A conceptual total-body scanner based on this technology, the 3Dπ, has demonstrated a simulated TOF resolution of 151 ps and an exceptionally high system sensitivity of 373 kcps/MBq, validating the immense potential of this approach.

While TOF technology significantly enhances the SNR, another key advancement—Depth of Interaction (DOI) information technology—emerged to address a different limitation. When gamma photons enter a long crystal at an oblique angle, a phenomenon known as the parallax effect occurs, in which the estimated LOR deviates from its true position. This effect is particularly pronounced at the edges of FOV, leading to a degradation in spatial resolution. DOI can effectively enhance spatial resolution and reduce parallax effects, particularly for positions away from the center of the radial FOV. A variety of methods have been developed for DOI acquisition, including signal waveform signal waveform discrimination (63, 64), spectral splitting (65, 66), dual-ended readout (67, 68), and innovative multilayer stacked scintillator arrays. For example, Binder et al. (69) employed interleaved triple-layer stacked LYSO scintillator array detectors with DOI for high-resolution and high-sensitivity small animal PET scanners. Ullah et al. (70) proposed a DOI detector utilizing scintillator emission wavelength, achieving excellent discrete DOI positioning accuracy with a DOI-separation FOM value of 1.5.

Currently, the state of the art small animal PET scanners—such as Albira Si, HiPET, SIAT aPET and Beta-CUBE (71)—as well as dedicated brain PET scanners like the NeuroExplorer and SIAT bPET (72), have universally integrated DOI measurement technology to ensure uniformly high spatial resolution across the entire FOV. For instance, the ultra–high-resolution brain PET developed by Zeng et al. (73) achieved an average spatial resolution of 1.53 mm FWHM.

In addition to detector optimization, innovations in the overall geometric configuration of the scanner also play a crucial role in enhancing performance. The traditional ring-shaped design, while widely adopted, is not necessarily the optimal solution. For instance, helmet-type brain PET and cubic brain PET designs, which are wearable and cover the entire brain, exhibit exceptional sensitivity and spatial resolution (74). The high-performance brain-dedicated PET scanner developed by Barrio et al. (75) features both high resolution and sensitivity. This design consists of an elliptical cylinder that conforms to the shape of the human head, with additional front and back panels to increase coverage. It achieves a sensitivity of up to 23% in cortical regions and 16% at the center of FOV. Such optimized geometric configurations better accommodate local imaging needs, resulting in enhanced imaging quality and accuracy.

Moreover, integrating high-resolution virtual-pinhole PET devices into long-axial-FOV scanners can substantially enhance imaging performance, markedly improving both the detectability and contrast of small lesions (76).

### 2.3 Compton camera

The Compton camera is a technology that utilizes the Compton effect for gamma-ray detection and imaging. Its most prominent advantage is the absence of mechanical collimation and the large imaging FOV. The Compton effect refers to the scattering of incident photons with atomic electrons in a substance, where photons scatter in different directions, resulting in different wavelengths, also known as Compton scattering. The Compton camera features a four-fold imaging FOV, enabling visualization of radiation sources with electronic collimation (77, 78). Based on the principle of Compton scattering, incoming gamma rays first undergo Compton scattering within the detector before experiencing the photoelectric effect. If the position and energy of each interaction are known, the Compton scattering angle can be calculated using the dynamic formula associated with the Compton effect. Lines connecting two interaction positions form the axis of a cone, with the angle derived from the scattering angle. Gamma-ray events must originate from the surface of this cone. As multiple such events are measured, the cones begin to converge at the true source position. In comparison to conventional modalities such as SPECT and SPECT/CT, which have demonstrated high diagnostic accuracy in specific clinical scenarios like unilateral condylar hyperplasia (UCH), the Compton camera represents a fundamentally different approach to gamma-ray imaging. While its design offers distinct advantages in terms of sensitivity and FOV, further research is required to validate its clinical applicability in UCH and other localized functional abnormalities (79).

Early Compton camera detectors utilized scintillation crystals; however, later models increasingly adopted semiconductor materials, particularly CZT, due to their high energy resolution and higher atomic number, which enhances susceptibility to Compton scattering. Compared to silicon materials, CZT offers greater detection efficiency, and its suitability for operation at room temperature overcomes the limitation of germanium detectors, which require low-temperature conditions.

Gunma University in Japan (80) has employed Si/CdTe Compton cameras for imaging radiopharmaceuticals such as ^99m^Tc and ^18^F. They successfully achieved *in vivo* tracer imaging in mice and conducted clinical trials. In 2019, Peng et al. (81) proposed the compton-PET system, based on GAGG-SiPM pixelated detectors. They incorporated a thin detector layer into the PET system as a scattering layer to form a Compton imaging system, thereby enhancing the sensitivity of the entire imaging system. Another innovative approach in Compton camera design is the SiFi-CC proposed by the research group led by Jonas Kasper et al. (82). It combines SiPMs with Compton cameras based on scintillating optical fibers. Both the scatterer and absorber are composed of elongated fibers made of high-density inorganic scintillating materials. This design exhibits a relatively low event pile-up rate in the detector, demonstrating high detection efficiency and rate capability due to its high granularity. Building on these advancements, recent prototypes such as the MACACO III Compton camera have further validated the feasibility of Compton-based imaging systems in clinical nuclear medicine. MACACO III successfully imaged both ^18^F-FDG and ^131^I tracers in phantom and patient studies, achieving spatial resolution superior to conventional gamma cameras and confirming its potential for radiopharmaceutical applications (83). In parallel, Compton cameras employing GAGG scintillators have also shown promise in prompt gamma imaging during proton therapy, enabling real-time visualization of beam shape and range estimation. This non-invasive verification method highlights the expanding clinical role of gamma imaging technologies beyond conventional diagnostics (84).

## 3 The development of multimodal imaging technology

Multimodal imaging technology has emerged as a significant focus in the field of radionuclide imaging, driven by the need for more comprehensive and accurate diagnostic tools. By integrating the complementary strengths of various imaging techniques, this technology enables the simultaneous acquisition of both functional metabolic and anatomical structural information in a single scan. This integration not only provides more comprehensive imaging data for disease diagnosis and treatment but also enhances the accuracy and reliability of imaging assessments, creating a synergistic effect where “1 + 1 > 2.”

### 3.1 CT-based multimodal fusion systems

The development of multimodal imaging technology began with the need to solve the challenge of registering functional images (e.g., SPECT) with anatomical images (e.g., CT) (85, 86). Before the advent of integrated hardware, researchers primarily relied on retrospective software-based image registration to fuse data from separate scanners, a process often complicated by inconsistencies in patient positioning, scan orientation, and image parameters between acquisitions. One of the pioneering efforts in SPECT/CT hardware fusion was accomplished by Hasegawa and his team at the University of California, San Francisco (UCSF). They configured a combined SPECT/CT prototype system by linking a clinical CT scanner (GE 9800) and a SPECT system (GE 600 XR/T) with a common patient table. This design allowed the patient to undergo both CT and SPECT scans in a single session via simple table translation, ensuring consistent patient geometry between the two datasets. The primary goal of this early work was not only to fuse functional and anatomical images but, more importantly, to use the high-resolution anatomical information from CT to perform accurate quantitative corrections for the SPECT data, such as patient-specific attenuation correction based on a CT-derived attenuation map and compensation for partial volume effects. This foundational research paved the way for subsequent commercial integrated SPECT/CT systems, the first of which became available for clinical use around 1999–2000 (87, 88).

SPECT/CT combines the functional imaging capabilities of SPECT with the anatomical imaging of CT, making it an advanced nuclear medicine imaging device (89). This hybrid system addresses a critical limitation of conventional planar scintigraphy and standalone SPECT—namely, the inability to precisely localize lesions within anatomical structures. In traditional SPECT applications such as benign and malignant bone diseases (90), thyroid cancer (91), neuroendocrine tumors, parathyroid adenomas, and sentinel lymph node (SLN) mapping of the head, neck, and pelvis regions (92), the anatomical context offered by CT substantially enhances diagnostic accuracy and clinical confidence (93). For example, in the diagnosis of pulmonary embolism (PE), ventilation/perfusion (V/Q) imaging combined with CT angiography demonstrates significantly higher diagnostic performance than standalone V/Q SPECT or Q SPECT imaging (94, 95). Clinically, SPECT/CT remains a mainstream tool for diagnosing coronary artery disease due to its widespread use in myocardial perfusion imaging and its favorable cost-effectiveness.

Major medical equipment companies such as GE, Philips, and Siemens Healthcare have introduced their own SPECT/CT systems. These systems integrate dual-head SPECT and CT scanners, allowing imaging on the same platform, significantly improving scanning efficiency and image quality. For example, GE Healthcare’s NM/CT 860 system has a scan time of less than 4 min, while Philips’ Precedence™ system completes a whole-body scan in less than 60 s. However, variations in system design also introduce specific clinical trade-offs. Another Philips SPECT/CT system, the BrightView XCT, combines a dual-head SPECT camera with a low-dose cone-beam CT. While it excels in anatomical high-resolution localization (0.33 mm isotropic voxels) and high-quality attenuation correction, offering potential benefits such as reduced artifacts and shorter examination times, its key limitation lies in the relatively slow acquisition process. As a result, it is not suitable for dynamic contrast-enhanced diagnostic examinations or for use as a conventional CT scanner. Siemens Healthcare’s Symbia™TruePoint SPECT/CT combines variable-angle dual-detector SPECT with Emotion™CT scanners in four different configurations, utilizing multi-detector CT components to obtain diagnostic-quality images. Despite the increasing technological maturity of SPECT/CT systems, their high cost continues to limit widespread adoption. Therefore, clinical decision-making must balance diagnostic benefits against cost-effectiveness. For instance, in the diagnostic pathway for UCH patients, SPECT alone is recommended as the primary imaging modality, as its diagnostic performance is sufficient while avoiding the potential drawbacks associated with SPECT/CT (79).

Despite these limitations, recent advancements in SPECT/CT—such as digital detectors, multidetector 3D geometries, and whole-body quantitative imaging—are further enhancing its diagnostic and quantitative capabilities, aiming to narrow the performance gap with PET. These innovations not only enhance image quality but also expand its utility in theranostics, particularly in biodistribution assessment and dosimetry planning for radionuclide therapy. Despite ongoing limitations in resolution and sensitivity, the modality retains clinical value, especially for imaging long-lived isotopes and performing multi-tracer studies. In addition, 4D SPECT/CT has demonstrated improved localization accuracy in complex cases such as hyperparathyroidism, offering a cost-effective, lower-radiation alternative to more conventional imaging options (96, 97).

Similar to SPECT, PET, lacking anatomical parameters to identify molecular events related to anatomy, is often integrated with Computed Tomography (CT) devices to form PET/CT systems. CT provides clear anatomical structure information, thereby enhancing the specificity and sensitivity of PET detection (98–101).

As a cornerstone of oncologic diagnosis, PET/CT has undergone continuous technological evolution aimed at improving scanning efficiency and quantitative accuracy. For example, the first continuous bed motion (CBM) PET/CT scanner introduced by Siemens AG, akin to spiral CT scanning, eliminates overlapping bed acquisitions. This system allows for flexible selection of the start and end positions for each body region within the PET scan coverage area, optimizing local sensitivity and improving axial noise uniformity. The sensitivity of PET/CT systems can be effectively increased with long axial FOV scanners that utilize silicon photomultipliers (SiPMs) detection systems. The 194 cm FOV scanner developed by Badawi et al. (102) significantly enhances sensitivity compared to standard axial FOV systems (103). The clinical significance of this progress is substantial. For example, the world’s first Biograph Vision Quadra PET/CT system from Siemens Healthineers features a FOV of 106 cm, while the standard axial FOV measures 26.3 cm. This scanner boasts a sensitivity of 174 cps/kBq and a time-of-flight (TOF) resolution of 219 ps (104), marking a 74% increase in sensitivity compared to PET devices using similar technology (105). However, the inherent limitations of PET/CT are also well recognized: its relatively high ionizing radiation exposure restricts its use in pediatric populations or patients requiring frequent follow-up, and its soft-tissue contrast resolution is intrinsically inferior to that of magnetic resonance imaging (MRI).

In multimodal imaging systems, the quality of CT images plays a pivotal role in determining overall diagnostic performance. The presence of metallic implants within the scanning field of view often leads to severe CT artifacts (such as streaks and shadows) that degrade image quality, obscure accurate assessment of tissues adjacent to metal objects, and potentially compromise CT-based attenuation correction and radiotherapy treatment planning accuracy. To address these challenges, various metal artifact reduction (MAR) algorithms have been developed and implemented clinically. These methods mitigate artifacts by correcting either projection data or reconstructed image data, thereby enhancing diagnostic reliability and improving treatment planning precision in regions near metallic implants. However, conventional MAR techniques are not without limitations; they may introduce secondary artifacts or distort the apparent size and shape of the metallic implants, and their effectiveness can vary depending on the algorithm type and implant material.

In recent years, deep learning–based reconstruction (DLR) has emerged as a promising artificial intelligence–driven approach in CT image reconstruction (106). DLR techniques have demonstrated great potential to produce images with lower noise and higher spatial resolution, while also showing advantages in artifact suppression and enabling reduced radiation dose scanning. Although the application and reliability of DLR in metal artifact correction remain under investigation, this approach represents a highly promising direction for improving CT image quality and, consequently, enhancing the overall performance of multimodal imaging systems.

### 3.2 Exploration of emerging and advanced multimodal imaging technologies

Driven by the growing demand for non-ionizing radiation imaging and superior soft-tissue resolution, PET/MRI emerged, representing the advanced frontier of multimodal imaging. Compared to CT, magnetic resonance imaging (MRI) offers advantages such as non-ionizing radiation, excellent soft tissue resolution, multiple sequence acquisitions, and high spatial resolution. PET/MRI (65, 107, 108) combines the ability of MRI to detect tissue density in soft tissues with the molecular detection capabilities of PET, enabling non-invasive, simple, and accurate acquisition of arterial input functions, thus achieving precise quantification of cerebral blood flow (CBF). Leveraging these strengths, PET/MRI is clinically positioned primarily for tumor and brain imaging, particularly in radiation-sensitive pediatric cases (109). However, a significant drawback of PET/MRI, when compared to PET/CT, is its substantially higher cost. Additionally, the MR electromagnetic field may interfere with pacemakers, defibrillators, or other implantable devices that are susceptible to electromagnetic interference, limiting its use in certain patient populations.

Several research groups are actively working on developing various configurations of MRI-compatible PET systems. Systems from different manufacturers (or vendors) reflect various design trade-offs, for instance, Siemens’ mMR system, based on the Verio MR scanner (110), incorporates PET detectors within the MR scanner, featuring a PET axial FOV of 25.8 cm and a photon sensitivity of 2.47%, however, its disadvantage is the lack of TOF capabilities. In contrast, GE’s Signa PET/MR (111) utilizes silicon photomultipliers (SiPMs) for TOF, achieving a resolution of 400 ps when the radiofrequency coil (RF) is open and 390 ps when closed, with a PET axial FOV of 25 cm and a photon sensitivity of 2.1%. In addition to Siemens and GE, United Imaging Healthcare is also a key participant in this field. Its uPMR 790 system utilizes a 32 cm axial FOV with digital Time-of-Flight (TOF) PET technology, achieving an industry-leading 2.8 mm NEMA spatial resolution (112). The system’s technology, derived from the uEXPLORER^®^, not only enhances routine clinical imaging capabilities, but its uSync research platform also provides new opportunities for advanced research, such as simultaneous cardiac PET/MR and functional neurological PET/MR.

Similarly, SPECT/MRI offers lower ionizing radiation doses and better imaging of soft tissues. The Hamamura group (113) designed a miniaturized dual-modality SPECT/MRI system, integrating CZT nuclear radiation detectors into a 4 T MRI system, enabling simultaneous acquisition of SPECT and MRI images. An important challenge facing SPECT/MRI is the system’s magnetic compatibility. To address this issue, Hutton et al. (114) developed the first MRI-compatible clinical SPECT system. The system consists of 20 MRI-compatible CsI(TI) detectors and a multi-slit collimator (115). The scanner achieves a spatial resolution of 8 mm (20 cm D × 9 cm L) in the FOV and a sensitivity of 0.036%. Additionally, Xiaochun et al. (116) studied a magnetic resonance-compatible SPECT system for simultaneous dual-mode imaging in small animals, achieving a spatial resolution of 350 μm and an energy resolution of 3–4 keV. Although still in an exploratory stage, the development of these technologies provides strong support for the application of SPECT/MRI and opens up new possibilities for clinical medicine.

In addition to fusion with anatomical imaging, fusion between functional imaging modalities is also being explored. SPECT/PET molecular imaging represents an innovative multimodal imaging approach that enables simultaneous visualization of positron-emitting and single-photon-emitting nuclides (117, 118). Several dual-head SPECT products currently available on the market also incorporate PET functionality, with some systems capable of simultaneously utilizing high-energy 511 keV radioactive nuclides for SPECT/PET imaging (119).

Combining Compton cameras with PET technology offers a promising solution for overcoming the limitations of PET in utilizing various radioactive tracers, particularly due to its suitability for multi-tracer imaging and high-energy radioactive tracer imaging. This integration allows for the simultaneous evaluation of different radioactive tracers under consistent conditions, thereby reducing errors associated with physical factors. Oganede et al. (120) developed a Compton-PET hybrid camera capable of simultaneously imaging single gamma rays and annihilation radiation emitted by diagnostic and therapeutic nuclides. This system can image each nuclide in the presence of different isotopes and switch between Compton and PET modes by optimizing energy windows. Similarly, Kim et al. (121) created a Compton imaging and PET coincidence system designed to monitor moving radioisotopes, leveraging Compton imaging to achieve a larger FOV. Yoshida et al. (122) proposed a concept known as whole gamma imaging (WGI), which utilizes all detectable gamma rays for imaging, resulting in direct imaging with excellent outcomes. Additionally, Omata et al. (123) developed a system capable of simultaneously performing three modes—Compton, pinhole, and PET—enabling the concurrent imaging of ^137^Cs, ^22^Na, and ^241^Am within the same FOV.

The emergence of tri-modal and quad-modal imaging systems has significantly expanded the scope and possibilities of imaging technology. Siemens’ Inveon system, a tri-modal imaging platform, integrates PET, SPECT, and CT modalities. It can be equipped with various parallel-hole, single-pinhole, or multi-pinhole collimators for whole-body and brain imaging in mice, achieving sub-millimeter spatial resolution. Similarly, Gamma Medica’s Triumph Trimodality scanner combines LabPET (PET), X-SPECT (SPECT), and X-O (CT) modes, specifically designed for preclinical and biomedical research applications in small animals. Wei et al. (124) developed a high-performance integrated animal PET/SPECT/CT system capable of performing tri-modal imaging of animals. Additionally, fluorescence molecular tomography (FMT), as a novel optical molecular imaging technique, has also been applied in multi-modal systems. The imaging principle of FMT involves injecting fluorescent substances into the subject, externally exciting fluorescence with light, collecting fluorescence photon information with detectors, and reconstructing images. Zhou et al. (125) developed a novel quad-modal small animal molecular imaging device, integrating CT, PET, SPECT, and FMT in the same system, enabling simultaneous multimodal imaging of the subject. The emergence of these multi-modal systems will provide more comprehensive and diversified imaging capabilities for scientific research and clinical applications, potentially leading to greater breakthroughs in disease diagnosis and treatment.

## 4 Innovative radionuclide imaging technology

### 4.1 Self-collimation

Self-collimation technology is an innovative approach to enhancing SPECT imaging performance. Traditional collimators are crucial components of SPECT, used for spatial localization selection (126, 127). However, mechanical collimators—typically made from heavy metals like lead or tungsten—absorb over 99.90% of photons during imaging, limiting both resolution and detection efficiency. Although methods such as multi-pinhole collimation and increased coverage solid angle have been implemented to improve traditional SPECT systems, their sensitivity remains significantly constrained by the reliance on physical collimators. The introduction of self-collimation technology effectively addresses this limitation, leading to improved imaging performance in SPECT systems (128).

Ma et al. developed a self-collimating SPECT system (128–131), in which the detectors themselves act as collimators. Arranged in a sparse array configuration within three-dimensional space, the detectors allow gamma photons to naturally collimate through absorption on their units. This design eliminates the need for traditional mechanical collimators. By achieving high spatial resolution without the significant photon loss associated with mechanical collimators, it overcomes the limitations of conventional systems and ensures high detection efficiency (128). For instance, a simulated human brain SPECT system utilizing this technology achieved an average sensitivity of 3.88%, which is more than an order of magnitude higher than conventional clinical SPECT systems that typically have sensitivities below 0.1%. This approach fundamentally resolves the trade-off between spatial resolution and detection efficiency caused by mechanical absorption collimation, resulting in a significant breakthrough in SPECT performance.

Building on this principle, Tsinghua University has developed a high-performance cardiac SPECT system (132) and a self-collimating SPECT system for small animals. The small animal self-collimating SPECT employs LYSO/GAGG composite detectors (133), with detector units measuring 1.35 × 2.7 × 7 mm^3^, providing high sensitivity to 140 keV gamma photons and achieving an inherent spatial resolution of 1.35 mm in one direction. The enhanced sensitivity and spatial resolution in the outermost layer of detectors significantly improve the overall imaging performance of the system. Guo et al. (134) conducted simulations on a whole-body self-collimating SPECT system and proposed a design for single-photon emission breast tomography (SC-SPEBT) based on self-collimation (135). SC-SPEBT consists of six detector panels that form a cylindrical FOV of 180 mm × 160 mm. The imaging system offers a resolution of 3 mm and a detection efficiency of 4.8%. Compared to planar molecular breast imaging, lesion detectability is improved. These studies underscore the significant potential of self-collimation technology in enhancing the performance of SPECT systems.

### 4.2 Cascade gamma photon imaging technique

Cascade radiation refers to the phenomenon in which a nucleus undergoes transitions from higher to lower energy levels during a single decay event, emitting two or more gamma photons of specific energies in quick succession. The cascade radiation photon pairs provide significantly better localization information about the nucleus compared to single-photon events in SPECT. Currently, the most commonly used radionuclides for cascade gamma imaging research are ^111^In (136, 137) and ^177^Lu (138–140). However, conventional PET imaging in nuclear medicine cannot image cascade radionuclides. SPECT relies solely on physical collimators and is unable to directly locate the position of nuclear decay or utilize the characteristics of cascade decay, thus limiting its ability to fully exploit the imaging potential of cascade radionuclides (141, 142). As a result, current clinical equipment in nuclear medicine has certain limitations regarding cascade radiation and cannot be used effectively (143). In recent years, advancements in detector and collimator technologies have led to a surge in research on cascade gamma photon coincidence imaging.

The detection methods and related performance of some existing cascade gamma photon coincidence imaging systems are shown in Table 1. In 2018, Pahlka et al. (144) from the M.D. Anderson Cancer Center in Texas, USA, designed the system for cascade gamma photon coincidence imaging with the spatial resolution better than 7.2 mm, but the detection sensitivity was only 6 × 10^−8^. The Mizuki research group (145–147) from the University of Tokyo verified the feasibility of cascade gamma photon coincidence imaging using the Compton imaging principle. The system achieved a high coincidence detection sensitivity of 3.57 × 10^−6^, but the spatial resolution is only 28 mm. In 2020, Chiang et al. (148) from Chang Gung University in Taiwan combined TOF technology with positron emission tomography (PET) to achieve collimation without a traditional collimator. The detection sensitivity for coincidences was approximately 2 × 10^−3^, but the spatial resolution was only 20 mm. Liu et al. (149, 150) from the Department of Physics at Tsinghua University proposed a real-time imaging cascade gamma photon coincidence imaging system, achieving a coincidence detection sensitivity of 3.4 × 10^−6^ and a spatial resolution of approximately 7.0 mm.

**Table 1:** Performance comparison of various detection methods for cascade gamma photon coincidence imaging systems.

Detection scheme	TOF-PET	GAGG-SiPM Compton imaging	Parallel-hole collimator with dual NaI detectors	Parallel-hole collimator with GAGG-SiPM
Current research status	Monte Carlo simulation	Experimental modules	Monte Carlo simulation	Experimental modules
Coincidence efficiency	2 × 10^−3^	3.57 × 10^−6^	6 × 10^−8^	~10^−8^
Spatial resolution	20 mm	28 mm	7.2 mm	4.1 mm
Energy resolution	12–18%@511 keV	12.4%@662 keV	Null	10.2%@245 keV

To understand the practical significance of these performance values (such as a spatial resolution of ~7.0 mm), the key is to compare them with the performance of standard SPECT systems. According to recent reviews, the collimator is the primary determinant of extrinsic spatial resolution in clinical SPECT, with typical values ranging from 6 to 15 mm at a 10 cm source-collimator distance (44). From this perspective, the best spatial resolution achieved by an experimental cascade imaging system (~7.0 mm) falls within the higher end of the typical clinical SPECT performance range. However, a more significant challenge lies in detection sensitivity. The reported coincidence sensitivities for these prototype systems, ranging from 10^−8^ to 10^−6^, are several orders of magnitude lower than those of conventional clinical systems. This low sensitivity is a primary barrier to acquiring images with adequate statistics in a clinically feasible scan time and represents the main hurdle for clinical translation.

Based on the analysis above, it is evident that while there have been advancements in cascade gamma photon coincidence imaging technology in recent years, a significant gap remains before these systems can be applied clinically. There is a strong demand in the field of radionuclide imaging for the development of high-performance, novel systems specifically designed for cascade gamma photon radiation detection and imaging.

## 5 Summary and prospect

This paper systematically reviews the technological advancements in the field of radionuclide imaging over the past decade, covering performance breakthroughs in traditional imaging (PET and SPECT), the evolution of multimodal fusion systems, and the exploration of cutting-edge technologies such as self-collimation and cascade gamma photon imaging. A review of these developments reveals that the core driving force of the entire field lies in continuously challenging the physical limits of imaging to meet the growing clinical demand for precision diagnosis and therapy. However, the path from technological innovation to clinical application remains challenging.

Looking ahead, the development of radionuclide imaging technology will be characterized by several key trends.

The first major trend is the deepening and maturation of theranostics, which currently represents the most promising development direction in nuclear medicine. The core of this strategy lies in using radiopharmaceuticals that possess both diagnostic imaging and targeted therapeutic functions to achieve precise disease assessment and personalized treatment. For example, radionuclides like ^177^Lu and ^90^Y emit both therapeutic *β*-rays and medium-energy *γ*-photons suitable for SPECT imaging during their decay. This dual capability holds significant promise for simultaneous therapy and imaging, presenting vast prospects for theranostic applications (151). The tremendous success of ^177^Lu-based drugs in treating neuroendocrine tumors and prostate cancer in recent years has fully demonstrated the clinical value of this paradigm (152, 153). To fully unleash the potential of theranostics, challenges must still be overcome. Future research will focus on a deeper understanding of the physical properties of complex radionuclides, such as non-pure positron emitters, to develop more agents with excellent theranostic characteristics (154). Meanwhile, accurate in-vivo dosimetry is key to personalized therapy. This not only requires imaging systems capable of precise quantification of therapeutic radionuclides but also leverages advanced algorithms like artificial intelligence—for instance, using deep learning to generate attenuation maps for improved SPECT quantification (155)—to establish a more precise, closed-loop theranostic workflow.

The second major driver is the leap from qualitative assessment to absolute quantification. With improvements in system performance and the refinement of correction algorithms, the future of radionuclide imaging will fully embrace an era of “quantification.” This implies moving beyond semi-quantitative metrics like the standardized uptake value to achieve absolute quantification of radiopharmaceutical concentration within lesions. This will enable the precise assessment of tumor heterogeneity, monitoring of early therapeutic response in small lesions, and provision of critical data for drug development.

Furthermore, artificial intelligence (AI), particularly deep learning, is set to permeate every aspect of radionuclide imaging. Beyond the well-researched application of CT-free attenuation correction in SPECT (156), AI will also play a critical role in multiple other domains, including image reconstruction and denoising (157), image analysis and diagnostic support (158), and workflow optimization. Examples include developing intelligent reconstruction algorithms capable of processing low-dose, fast-scan data, thereby further reducing patient radiation dose and examination time while maintaining image quality. AI will also be used to automatically identify, segment, and quantify lesions, providing more stable and reproducible quantitative results, and to construct predictive models based on multimodal imaging data to aid in clinical decision-making. Additionally, it will automate scanning protocols and image post-processing workflows, enhancing equipment operational efficiency.

Finally, the continuous exploration of novel detectors and system designs persists. The endless pursuit of higher-performance detector materials will continue to drive breakthroughs in the temporal, energy, and spatial resolution of imaging systems. Simultaneously, dedicated high-resolution imaging systems for specific clinical needs (such as brain or breast imaging), as well as ultra-high-sensitivity systems capable of total-body coverage, will open new avenues for systems biology research and ultra-early disease screening.

In summary, radionuclide imaging is transitioning from a field primarily focused on morphological and functional observation to a modern medical imaging discipline centered on precise quantification, multidimensional information fusion, and integrated diagnostics and therapy. Future breakthroughs will depend not only on innovations in physics and engineering but also on deep interdisciplinary integration with clinical medicine, pharmacology, and data science to ultimately achieve a deeper understanding of diseases and more effective interventions.
